# Conformational state interactions provide clues to the pharmacochaperone potential of serotonin transporter partial substrates

**DOI:** 10.1074/jbc.M117.794081

**Published:** 2017-08-23

**Authors:** Shreyas Bhat, Peter S. Hasenhuetl, Ameya Kasture, Ali El-Kasaby, Michael H. Baumann, Bruce E. Blough, Sonja Sucic, Walter Sandtner, Michael Freissmuth

**Affiliations:** From the ‡ Institute of Pharmacology and the Gaston H. Glock Research Laboratories for Exploratory Drug Development, Center of Physiology and Pharmacology, Medical University of Vienna, A-1090 Vienna, Austria,; the § Translational Pharmacology Section, Intramural Research Program, National Institute on Drug Abuse, Baltimore, Maryland 21224, and; ¶ Center for Drug Discovery, Research Triangle Institute, Research Triangle Park, North Carolina 27709-1294

**Keywords:** chaperone, dopamine transporter, electrophysiology, serotonin, serotonin transporter

## Abstract

Point mutations in SLC6 transporters cause misfolding, which can be remedied by pharmacochaperones. The serotonin transporter (SERT/SLC6A4) has a rich pharmacology including inhibitors, releasers (amphetamines, which promote the exchange mode), and more recently, discovered partial substrates. We hypothesized that partial substrates trapped the transporter in one or several states of the transport cycle. This conformational trapping may also be conducive to folding. We selected naphthylpropane-2-amines of the phenethylamine library (PAL) including the partial substrate PAL1045 and its congeners PAL287 and PAL1046. We analyzed their impact on the transport cycle of SERT by biochemical approaches and by electrophysiological recordings; substrate-induced peak currents and steady-state currents monitored the translocation of substrate and co-substrate Na^+^ across the lipid bilayer and the transport cycle, respectively. These experiments showed that PAL1045 and its congeners bound with different affinities (ranging from nm to μm) to various conformational intermediates of SERT during the transport cycle. Consistent with the working hypothesis, PAL1045 was the most efficacious compound in restoring surface expression and transport activity to the folding-deficient mutant SERT-^601^PG^602^-AA. These experiments provide a proof-of-principle for a rational search for pharmacochaperones, which may be useful to restore function to clinically relevant folding-deficient transporter mutants.

## Introduction

Transporters for the monoamines serotonin (SERT),^3^ dopamine (DAT), and norepinephrine (NET) belong to the solute carrier-6 (SLC6) family. SLC6 transporters are examples of secondary active transporters that utilize the electrochemical gradient of sodium to drive cellular uptake of substrates. SERT, DAT, and NET work in relay with vesicular monoamine transporters to ensure efficient recycling of neurotransmitters from the extra-neuronal space (*i.e.* the synapse) into the synaptic vesicles. This effectively terminates signaling by the released neurotransmitter and replenishes synaptic stores (1). Monoaminergic neurons reside in the mesencephalon or rhombencephalon and project diffusely into many other brain areas including the cerebral cortex and the basal ganglia by elaborating dense axonal arborizations (2–4). Hence, monoamines function as neuromodulators and impinge on the wired transmission exerted for instance by glutamatergic projections. Volume transmission elicited by monoamines can be tuned by changing the activity of the monoamine transporters. Accordingly, SERT, DAT, and NET are prime targets for both therapeutically relevant and illicit drugs. Because the transporters are closely related, they share inhibitors and substrates. The illicit market provides lucrative incentives to explore the chemical space in the vicinity of the known ligands. This results in a very rich pharmacology of DAT, NET, and SERT (5). Exogenous ligands, which bind to monoamine transporters, are classified as inhibitors if they block neurotransmitter reuptake through the transporter (*e.g.* cocaine, tricyclic antidepressants, selective serotonin reuptake inhibitors/SSRIs) or as substrates/releasers; amphetamine-like releasers induce efflux of the endogenous monoamine because they are taken up into the cell via the transporter but they switch the transporter from the cyclical forward transport mode into the exchange mode (5). Irrespective of the mechanism of action, both releasers and inhibitors increase extracellular neurotransmitter levels and, hence, signaling via the cognate receptors. In the case of the dopamine transporter, the reinforcing and rewarding characteristic of these drugs lead to substance addiction. Consequently, any exogenous ligand that acts either as an inhibitor of, or as a releaser on DAT is predicted to have addictive properties. However, the discovery of atypical inhibitors and partial releasers has challenged this notion (6). Atypical inhibitors of DAT such as vanoxerine, modafinil, and benztropine have been shown to have far less reinforcing and psychostimulant effects than cocaine in people (7, 8). Similarly compounds have also been discovered that were classified as “partial substrates” because they induce neurotransmitter efflux with lower efficacy when compared with D-amphetamine (9, 10).

Understanding the pharmacology of such atypical ligands has an appealing application in the treatment of addiction disorders. In addition, they may be useful to correct the folding defect of transporter mutants by virtue of their pharmacochaperoning action (11). The folding trajectory of membrane proteins, in general, and of SLC6 transporters, in particular, is poorly understood, but it is clear that conformations are being visited that can be stabilized by ligands. This is exemplified by the plant alkaloid ibogaine and its derivative noribogaine, which are shown to trap SERT in the inward-facing conformation (12, 13) and to rescue folding-deficient mutants of SERT (14–17). In contrast, neither inhibitors such as imipramine nor substrates/releasers such as *p*-chloroamphetamine rescue folding-deficient versions of SERT (14). Based on these observations, we surmised that partial releasers ought to act as pharmacochaperones provided that they stabilize the transporter in a conformation conducive to folding. We tested this conjecture by analyzing the actions of “partial releasers” (9) on the transport cycle of SERT. Our analysis relied on electrophysiological recordings because these allow for estimating the affinity of compounds to various conformational intermediates in the transport cycle (18–21). This approach identified PAL1045 as a candidate pharmacochaperone, an action that was confirmed using a folding-deficient mutant of SERT.

## Results

### The naphthylpropane-2-amines PAL287, PAL1045, and PAL1046 differ in their inhibitory potency

**Figure 1.**
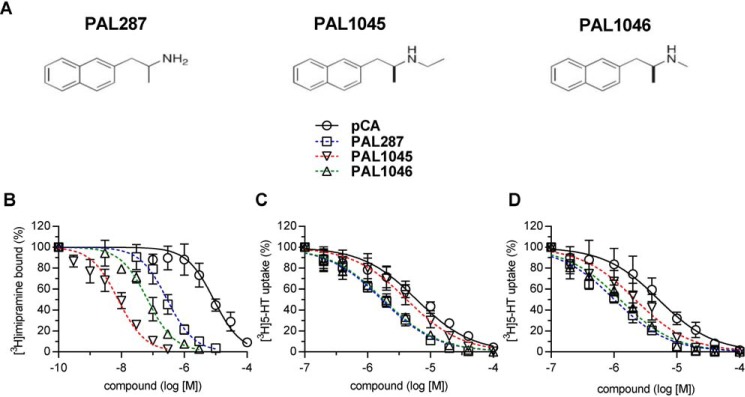
**Inhibition by PAL287, PAL1045, PAL1046, and *p*-chloroamphetamine of [^3^H]imipramine binding to and [^3^H]5-HT uptake by SERT.** *A*, chemical structures of PAL287, PAL1046, and PAL1045. *B*, membranes (2.5 μg/assay) were prepared from HEK293 cells, which stably expressed YFP-SERT, and incubated in the presence of [^3^H]imipramine (3 nm) and of the indicated concentrations of PAL287, PAL1045, PAL1046, and pCA for 1 h at 20 °C. Nonspecific binding, which was <10% of total binding, was determined in the presence of 10 μm paroxetine. Specific uptake (*B*) and specific binding (*C*) were 18.0 ± 3.8 pmol·min^−1^·10^−6^ cells and 51.7 ± 4.5 fmol/assay, respectively, and was set to 100% to normalize for inter-assay variation. *C* and *D*, HEK293 cells stably expressing wild-type YFP-hSERT were seeded onto 48-well plates for 24 h. Specific [^3^H]-5-HT uptake (0.2 μm) was measured over a 1-min reaction time either in the absence or presence of the indicated concentrations of PAL287, PAL1045, PAL1046, and pCA. In *panel D* cells were preincubated for 20 min with the inhibitors before initiating the uptake reaction by the addition of 0.2 μm ^3^[H]-5-HT. Paroxetine (10 μm) was used to determine nonspecific uptake, which was <5% of total uptake. Data are as the means ± S.D. (*error bars*) from five (*B*) and four (*C*) independent experiments done in triplicate (*B*) or duplicate (*C*). The curves were generated by fitting a sigmoidal function through data points normalized between 0 and 100% inhibition. The concentrations giving half-maximum inhibition (IC_50_ values) generated from each plot are stated in Table 1.

The PAL series of amphetamine-related compounds was previously examined for their ability to induce reverse transport. We selected three naphthylpropane-2-amines from this list because they differed in their ability to release substrate (9) and because they represent a series of congeners, *i.e.* PAL1046 and PAL1045 are the methylated and ethylated derivatives of PAL287 (Fig. 1*A*). PAL1045 was classified as a partial releaser, *i.e.* saturating concentrations of PAL1045 caused less substrate efflux than the full releaser D-amphetamine (9). Partial release can be explained by assuming that ineffective releasers lock the transporter in intermediate conformations during the transport cycle. This kinetic trap may impede efficient reverse transport. This hypothesis predicts that partial releasers bind with high affinity to SERT. Accordingly, we compared the potency of the three compounds to displace [^3^H]imipramine binding to membranes prepared from HEK293 cells expressing the human SERT with their potency to inhibit uptake of [^3^H]-5-hydroxytryptamine ([^3^H]5-HT) by these cells (Fig. 1*B*). The affinity of the reference compound *p*-chloroamphetamine was comparable in the two assays (*open circles* in Fig. 1, *B* and *C*, Table 1). In contrast, PAL287, PAL1045, and PAL1046 were substantially more potent in inhibiting [^3^H]imipramine binding (Fig. 1*B*) than cellular substrate uptake (Fig. 1*C*; Table 1). The most pronounced shift was observed with PAL1045, which was ∼500-fold more potent in displacing [^3^H]imipramine binding than in inhibiting substrate uptake (Table 1). With PAL287 and PAL1046, the ratios were smaller but still substantial, *i.e.* ∼6- and 26-fold, respectively (Table 1). Binding experiments were done under equilibrium conditions. In contrast, the reaction time was only 1 min in the uptake assay. We verified that the low apparent affinity observed in the uptake assay was not due to the short incubation time by preincubating cells with the compounds for 20 min before the addition of [^3^H]5-HT (Fig. 1*D*). However, this preincubation did not enhance the inhibitory potency of any of the compounds (*cf.* Fig. 1. *C* and *D*). Thus, the discrepancy between binding and uptake experiments presumably arises from the different assay conditions; during cellular uptake the transporter is allowed to cycle in the forward transport mode or to seesaw through the substrate-exchange mode in a manner driven by the ionic gradients. In contrast, no ionic gradient is imposed on SERT in the binding assay. Thus, the transporter can accumulate in a state in which it binds a given ligand with high affinity.

**Table 1:** **Affinity estimates for PAL287, PAL1045, PAL1046, and the reference compound *p*-chloroamphetamine** The IC_50_ for inhibition of [^3^H]5-HT uptake was calculated from the curves shown in Fig. 1*B*; because the substrate concentration was 0.2 μm and thus far below *K_m_*, IC_50_ ≅ *K_i_*. The *K_i_* for inhibition of [^3^H[imipramine binding was calculated from the IC_50_ values determined in Fig. 1*C* by correcting for the *K_D_* of the radioligand, which was determined by saturation binding. The bimolecular association rate constant *k*_on_ and the dissociation rate constant *k*_off_ were determined by electrophysiological recordings summarized in Figs. 3 and 4, respectively. Shown are the arithmetic means from 4 to 5 determinations; the numbers in parentheses are the 95% confidence interval. The kinetically derived dissociation constant *K_D_*_,kin_ was calculated from the ratio of *k*_off_/*k*_on_.

	IC_50_ (uptake inhibition)	*K_i_* (binding inhibition)	*k*_on_ (peak current relaxation rate)	k_off_ (peak current recovery rate)	*K_D_*,_kin_ (*k*_off_/*k*_on_)
	*nM*	*nM*	*10^6^m*^−*1*^ *s*^−*1*^	*s*^−*1*^	*nM*
*p*-Chloroamphetamine	6042 (5504-6633)	4880 (3382-5665)	0.74 (0.67-0.80)	3.53 (3.05-4.00)	4786
PAL287	1686 (1582-1798)	144 (134-175)	5.45 (3.81-7.10)	0.12 (0.10-0.14)	22.1
PAL1045	4212 (3782-4691)	4 (3-5)	4.67 (2.70-6.65)	0.026 (0.023-0.029)	5.5
PAL1046	1796 (1692-1907)	34 (29-40)	5.51 (4.29-6.73)	0.13 (0.11-0.14)	22.9

### Steady-state currents through SERT induced by PAL287, PAL1045, and PAL1046

**Figure 2.**
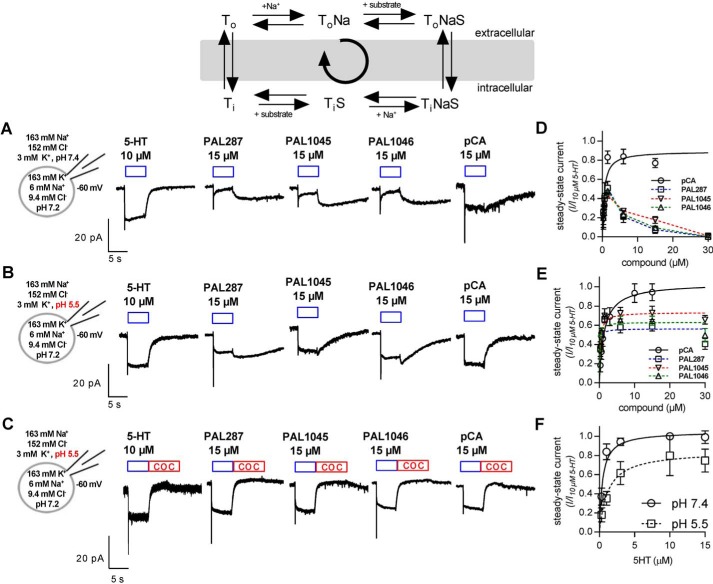
**Steady-state currents elicited by the application of PAL287, PAL1045, PAL1046, and pCA at pH 7.4 and pH 5.5.** The reaction scheme outlines the transport cycle were *T_o_*, *T_o_Na*, and *T_o_NaS* correspond to the transporter in the empty, sodium-bound, and (sodium+substrate)-liganded state, respectively, and T_i_, T_i_S, and T_i_NaS are the analogous inward-facing states. The initial peak current corresponds to the charge movement in the membrane electric field and tracks the conformational transitions required to move from T_o_Na to T_i_NaS. The steady-state current reflects the movement through the entire cycle. *A*, schematic representations of the experimental conditions and representative traces of steady-state currents generated by the application of the indicated compounds to HEK293 stably expressing GFP-tagged SERT in the presence of physiological ion gradients at a holding potential of −60 mV and at pH 7.4. *B* and *C*, the conditions were similar to *panel A* with the exception of pH 5.5 in the external solution (as opposed to pH 7.5). In *panel C*, 100 μm cocaine (*COC*) was washed in where indicated by the *red bar* to show that the slowly decay of the current in *panel B* was due to back diffusion of the lipophilic compounds (PAL287, PAL1045, PAL1046, and pCA). *D* and *E*, the amplitude of the steady-state current of SERT was determined at the indicated concentrations of PAL287, PAL1045, PAL1046, and pCA and normalized (as a ratio) to the current amplitude elicited by a pulse of 10 μm 5-HT in the same cell (*n* = 5 cells per substrate per condition). *F*, the steady-state current elicited by the indicated concentrations of serotonin were recorded at pH 7.4 and 5.5. Data are the means ± S.D. from four to eight independent recordings. Where reasonable, curves were drawn by fitting the data points to a rectangular hyperbola resulting in the following *K_m_* ± S.D.: *p*-chloroamphetamine 0.54 ± 0.13 and 1.34 ± 0.16 μm; 5-HT = 0.66 ± 0.12 and 1.06 ± 0.13 μm at pH 7.4 and 5.5, respectively; PAL287 0.11 ± 0.05 μm, PAL1045 0.27 ± 0.05 μm, and PAL1046 0.12 ± 0.04 μm at pH 5.5.

Electrophysiological recordings allow for probing the transport cycle of SERT; the substrate-induced current through the transporter is composed of two components: (i) an initial peak current (*cf.* the *left hand current trace* in Fig. 2*A*), which reflects the substrate-induced translocation of Na^+^ and thus monitors the transition from the outward to the inward-facing state (21); (ii) peak current followed by a sustained current, which persists as long as the cell is exposed to substrate (left hand current trace in Fig. 2*A*). This steady-state current corresponds to a Na^+^-conducting state, which is visited by the K^+^-bound inward-facing conformation during the rate-limiting return step. Hence, it is a read-out for completion of the transport cycle (18). Accordingly, the sustained current can be used to assess if a compound drives SERT into the forward transport mode; that is, if it is a substrate. It is evident from the representative traces shown in Fig. 2*A* that the steady-state currents elicited by PAL1045, PAL287, and PAL1046 were smaller than those elicited by 5-HT or *p*-chloroamphetamine (pCA). In addition, upon washout the steady-state current did not decay, but increased transiently. This is indicative of rapid intracellular accumulation of the compounds by diffusion during the superfusion and back diffusion during washout (20, 22). Accordingly, we reduced the extracellular pH to 5.5 to force protonation of the compounds and to thus suppress cell entry by diffusion. Under these conditions, the steady-state current elicited by PAL1045, PAL287, and PAL1046 was substantially larger than at pH 7.4 (*cf.* Fig. 2, *A* and *B*). In addition, upon washout, the current either did not increase (PAL1045 in Fig. 2*B*), or the increase was modest, when compared with recordings done at pH 7.4 (*cf.* PAL287 and PAL1046 in Fig. 2, *A* and *B*). Finally, if the inhibitor cocaine was washed in, the current decay was rapid and uniform regardless of whether 5-HT, PAL287, PAL1045, PAL1046, or pCA was examined (Fig. 2*C*). Accordingly, at pH 7.4, the concentration-response curve for PAL1045, PAL287, and PAL1046 was bell-shaped (Fig. 2*D*), but at pH 5.5 we observed a saturation hyperbola (Fig. 2*E*); *K_m_* values were 0.11 ± 0.05, 0.27 ± 0.04, and 0.12 ± 0.03 μm for PAL287, PAL1045, and PAL1046, respectively, and the maximum steady-state currents were between 55 and 72% that seen in the presence of saturating 5-HT concentrations. The *K_m_* of the reference compound *p*-chloroamphetamine was 1.34 ± 0.16 μm, and the maximum current elicited by saturating concentrations was 102 ± 4% that elicited by 10 μm serotonin. The drop in pH also had only a modest effect on the *K_m_* of serotonin when assessed by measuring the steady-state current (Fig. 2*F*) or cellular uptake (data not shown). Thus, the observations showed that PAL287, PAL1045, and PAL1046 were substrates of SERT but that they were transported less effectively than the natural substrate 5-HT or *p*-chloroamphetamine. This is consistent with their high affinity binding to one of the conformational intermediates in the transport cycle.

### Affinity estimates for binding of PAL287, PAL1045, and PAL1046 to the outward-facing conformation of SERT

**Figure 3.**
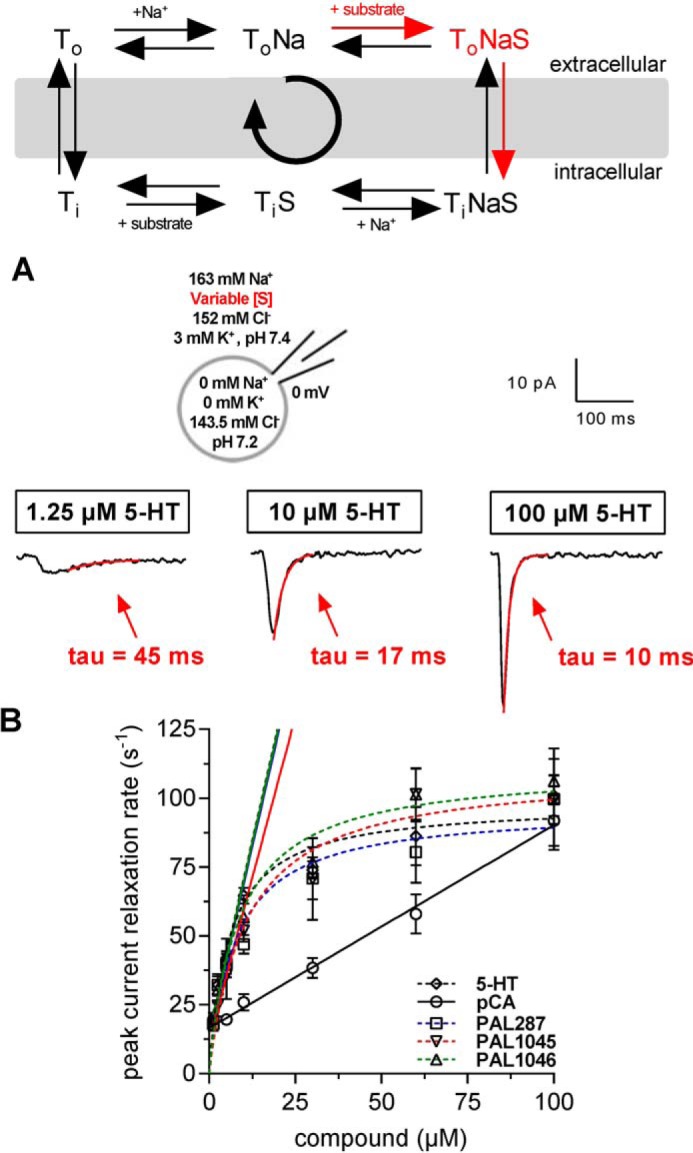
**Peak current relaxation rate of SERT as a function of ligand concentration.** *Top*: the *red arrows* in the reaction scheme (see Fig. 2 for abbreviations) outline the steps of transport cycle, which were probed by the recording condition. *A*, schematic representation of the experimental conditions (*left-hand scheme*) and representative traces of 5-HT induced peak currents when applied at 1.25, 10, and 100 μm at a holding potential of 0 mV. Each trace is representative of five independent experiments. The relaxation of peak current was fit to the equation of a mono-exponential decay to obtain the relaxation rate *k*_pr_ (τ = 1/*k*_pr_). *B*, the relaxation rate was determined at the indicated concentrations of serotonin/5-HT (♢), PAL287 (□), PAL1045 (▿), PAL1046 (▵), and pCA (○). Data points are the mean ± S.D. (*n* = 5) and were subjected to nonlinear least-squares curve-fitting to a hyperbola except for the data points of pCA, which were fitted by linear regression. For serotonin/5-HT, PAL287, PAL1045, and PAL1046, the association rate constant *k*_on_ was calculated from the slope of the regression line fitted to the linear portion, *i.e.* the first three points. These rates are compiled in Table 1.

The apparent affinities of PAL287, PAL1045, and PAL1046 differed substantially depending on whether they were examined for their ability to block substrate uptake (Fig. 1*C*) or inhibitor binding (Fig. 1*B*) or to serve as substrates (Fig. 2*D*). In contrast, the variation in affinity estimates was less pronounced for the reference compound *p*-chloroamphetamine. We examined the affinity of the naphthylpropan-2-amines to the outward-facing conformation of SERT by recording peak currents. The peak current results from the binding of substrate and co-substrate ions and the ensuing translocation of the charge through the membrane electric field, which is associated with the conformational transition from the outward- to the inward-facing conformation. The relaxation of this current reflects the charge movement (23); it is the first derivative of the position of the charge over time. Initially, the current is at a maximum, because all charges are on one side of the membrane electric field. When all charges have moved, the current subsides (it relaxes to 0). Hence, the relaxation rate comprises two reactions; that is, the apparent binding rate *k*_app_ of substrate (co-substrate Na^+^ is already present) and the charge movement proper. At low substrate concentrations the apparent association rate *k*_app_ is rate-limiting. At higher concentrations, the conformational transition required for charge movement became rate-limiting (see the reaction scheme in Fig. 3). The peak current can be studied in isolation by eliminating K^+^ from the intracellular solution because K^+^-binding to the inward-facing conformation of SERT is required for induction of the steady-state current (21). In the absence of intracellular Na^+^, SERT cannot enter into the exchange mode. Thus, under the experimental conditions employed in Fig. 3, the rate of peak current relaxation (*k*_pr_) reflects the rate of substrate binding (*k*_app,S_) and the rate of the conformational transition (*k*_t_), *i.e.* 1/*k*_pr_ = 1/*k*_app,S_ + 1/*k*_t_. It is evident that at low substrate concentrations, the apparent association rate of substrate is limiting for *k*_pr_: τ decreased with increasing concentrations of the substrate applied until at high substrate concentration the conformational transition of SERT rather than substrate-binding dictates *k*_pr_ (Fig. 3, *A* and *B*). For 5-HT, PAL287, PAL1045, and PAL1046, we did not find any appreciable difference in the relation between their concentration and the observed peak current relaxation rate. Accordingly, we deduced comparable association rate constants (*k*_on_) of ∼5 × 10^6^ × m^−1^ s^−1^ for 5-HT, PAL287, PAL1045, and PAL1046 from the linear relation between ligand concentration and apparent rate (in a second order reaction) by extracting it from the first derivative at 0 ligand concentration of the exponential function shown in Fig. 3*B* (see also Table 1). Surprisingly, *p*-chloroamphetamine bound much more slowly than all other compounds such that the peak current relaxation rate was dictated by its apparent rate of association up to 100 μm (□ in Fig. 3*B* and Table 1).

**Figure 4.**
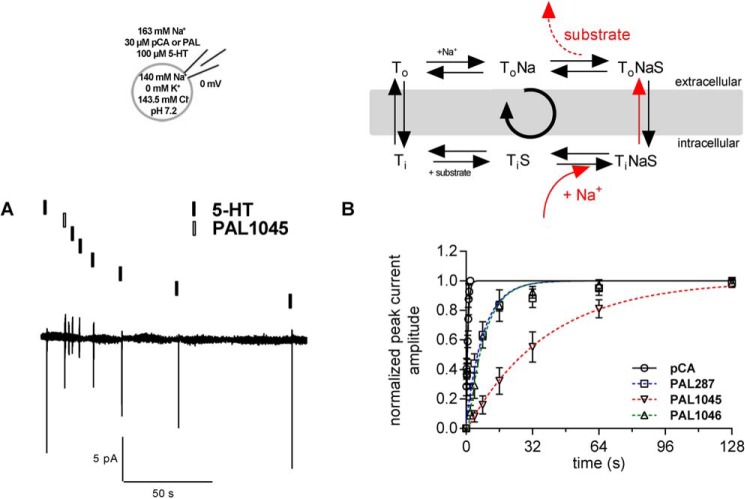
**Time course of peak current recovery in the presence of *p*-chloroamphetamine, PAL287, PAL1045, and PAL1046.** *Top*: schematic representation of the experimental conditions and of the transport cycle (see Fig. 2 for abbreviations), where the *red arrows* outline the steps of transport cycle, which were probed by the recording condition. *A*, peak recovery protocol and the corresponding current traces recorded at a holding potential of 0 mV in a representative experiment; a pulse of 5-HT (100 μm, *black box*) was applied to obtain a reference peak. Subsequently, a pulse of PAL1045 (30 μm, 200 ms; *white box*) was applied followed by increasing washout intervals, after which subsequent pulses of 5-HT were applied. Peak amplitudes after specific wash intervals represent the fraction of transporters available for 5-HT binding and thus recovery to the outward open state of SERT. *B*, the time course for 5-HT peak recovery was determined after application of PAL287 (□), PAL1045 (▿), PAL1046 (▵), and pCA ○) as outlined in *panel A*. The data (means ± S.D., *n* = 5) were fitted to the exponential for a mono-exponential rise to a maximum. The resulting constant represents the dissociation rate (*k*_off_) of the compound from SERT. Rates obtained for each compound are compiled in Table 1.

Dissociation rates were determined by recording peak current recovery under conditions where the forward transport mode was suppressed by the presence of high internal Na^+^ (140 mm). This condition also precludes substrate dissociation from the inward-facing conformation of SERT and forces SERT to operate in the substrate exchange mode (18, 21); after the initial conformational transition to the inward-facing state, the substrate-bound transporter oscillated between inward- and outward-facing conformation and released the bound substrate as a consequence of increasing superfusion intervals (see reaction scheme in Fig. 4). As a consequence of the dissociation, the number of unliganded SERT molecules increased over time. Hence, the peak current amplitude, which can be gauged by applying a pulse of 5-HT at a saturating concentration, is predicted to recover to its original value over time; a representative time course is shown in Fig. 4*A*, where the first (left hand) peak current was elicited by 100 μm 5-HT, the second pulse by 30 μm PAL-1045 (for 200 ms), and the subsequent currents by repeated challenges with 5-HT, whereas the cell was continuously superfused with buffer. It is evident from Fig. 4*A* that the recovery of the full-sized peak current required 128 s. A comparison of *p*-chloroamphetamine with the three naphthylpropane-2-amines revealed that the peak current recovered fast after *p*-chloroamphetamine (○ in Fig. 4*B*). The recovery was ∼18-fold slower for PAL-1046 (▵ in Fig. 4*B*) and for PAL-287 (□) in Fig. 4*B*) resulting in half-lives of ∼5.5 s (Table 1). The dissociation of PAL1045 (▿ in Fig. 4*B*) was even slower and proceeded with a half-life of ∼27 s (Table 1). The association and dissociation rate constants obtained from the experiments depicted in Figs. 3 and 4 allowed for the calculation of *K_D_* estimates. These were in reasonable agreement with the affinity estimates obtained from the binding experiments (Table 1).

**Figure 5.**
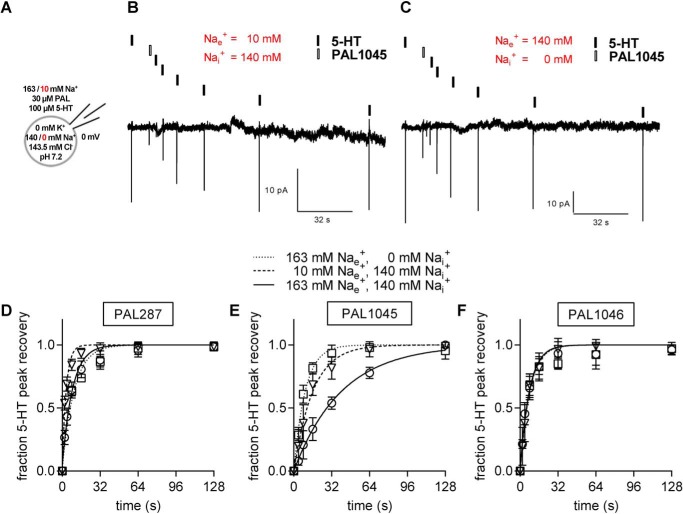
**Effects of variation in the internal and external Na^+^ concentration on the peak current recovery rate after exposure to PAL287, PAL1045, and PAL1046.** *A*, schematic representation of the experimental conditions, where extracellular and intracellular Na^+^ was varied to give three conditions, *i.e.* high extracellular (163 mm) and intracellular (140 mm) Na^+^, high extracellular (163 mm) and no intracellular (0 mm) Na^+^, and low extracellular (10 mm) and high intracellular (140 mm) Na^+^. *B* and *C*, protocol for time course of peak recovery (as outlined in Fig. 4) and representative traces under conditions of low extracellular and high intracellular Na^+^ (10 mm/140 mm; *panel B*) and high extracellular (140 mm) and no intracellular Na^+^ (140 mm/0 mm; *panel C*). *D–F*, the recovery rates were determined at high extracellular (163 mm) and intracellular (140 mm) Na^+^ (○), high extracellular (163 mm) and no intracellular (0 mm) Na^+^ (□), and low extracellular (10 mm) and high intracellular (140 mm) Na^+^ (▵) for PAL287 (*D*), PAL1045 (*E*) and PAL1046 (*F*). Data represent the means ± S.D. (*n* = 5). Under these three conditions the recovery rates *k*_rec_ (95% confidence intervals) were: for PAL 287: 0.116 s^−1^ (0.099–0.133 s^−1^), 0.100 s^−1^ (0.086–0.114 s^−1^), and 0.299 s^−1^ (0.264–0.335 s^−1^); for PAL1045: 0.024 s^−1^ (0.022–0.027 s^−1^), 0.105 s^−1^ (0.093–0.117 s^−1^); for PAL-1045: 0.062 s^−1^ (0.051–0.073 s^−1^); for PAL1046: 0.127 s^−1^ (0.105–0.150 s^−1^), 0.121 s^−1^ (0.099–0.142), and 0.125 s^−1^ (0.102–0.147 s^−1^).

It is obviously not possible to determine the apparent affinity of a compound to SERT in the total absence of Na^+^ by the available electrophysiological means because without Na^+^ there is no current to be recorded. However, the Na^+^ dependence of binding can be inferred from the effects seen in the presence of low Na^+^ concentrations. In the presence of 10 mm external Na^+^, the recorded currents were large enough to allow for an analysis of the rate of peak current recovery according to the protocol outlined in Fig. 4. We used this approach to examine the effect of lowering external Na^+^ on the dissociation rate of PAL287, PAL1045, and PAL1046 (*original traces* and *triangles* in Fig. 5*B* and *D–F*, respectively). In addition, we also recorded the rate of peak-current recovery after elimination of internal Na^+^ and compared the time course of recovery to that seen in the presence of high internal Na^+^ concentrations (*squares* and *circles* in Fig. 5, *D–F*, respectively). In the absence of internal Na^+^, the transporter can also release the compound on the intracellular side before its return to the outward-facing state. It is evident that the changes in Na^+^ concentrations had no appreciable effect on the peak current recovery rate *k*_rec_ after exposure of SERT to PAL1046 (Fig. 5*F*: *k*_rec_ = 0.127 s^−1^, confidence interval (CI) = 0.105–0.150 s^−1^ in the presence of high external and internal Na^+^; *k*_rec_ = 0.121 s^−1^, CI = 0.099–0.142 s^−1^ in the absence of internal Na^+^; *k*_rec_ = 0.125 s^−1^, CI = 0.102–0.147 s^−1^ in the presence of 10 mm external Na^+^). In contrast, lowering external Na^+^ accelerated the peak current recovery rate of PAL287 (Fig. 5*D*), *i.e.* at 10 mm external Na^+^ the dissociation rate (*k*_rec_ = 0.299 s^−1^; CI = 0.264–0.335 s-^1^) was 2.6× faster than at high (163 mm) external Na^+^ (*k*_rec_ = 0.116 s^−1^; CI = 0.099–0.133 s^−1^), but omitting internal Na^+^ did not affect the peak current recovery rate (τ = 0.100 s^−1^; CI = 0.086–0.114 s^−1^). Likewise after application of PAL1045, the peak current recovery rate also recovered 2.6-fold faster in the presence of low external Na^+^ (▿ in Fig. 5*E*, *k*_rec_ = 0.062 s^−1^; CI = 0.051–0.073 s-^1^) than in the presence of high external Na^+^ (circles in Fig. 5*E*, *k*_rec_ = 0.024 s^−1^; CI = 0.022–0.027 s^−1^). In addition, in the nominal absence of internal Na^+^, the dissociation of PAL1045 was also accelerated (□ in Fig. 5*E*; *k*_rec_ = 0.105 s^−1^; CI = 0.093–0.117 s^−1)^. Thus, in the nominal absence of Na^+^ the difference in affinity between PAL1045 and the other two compounds was eliminated.

### Dissociation of PAL287, PAL1045, and PAL1046 from the inward-facing state of SERT

**Figure 6.**
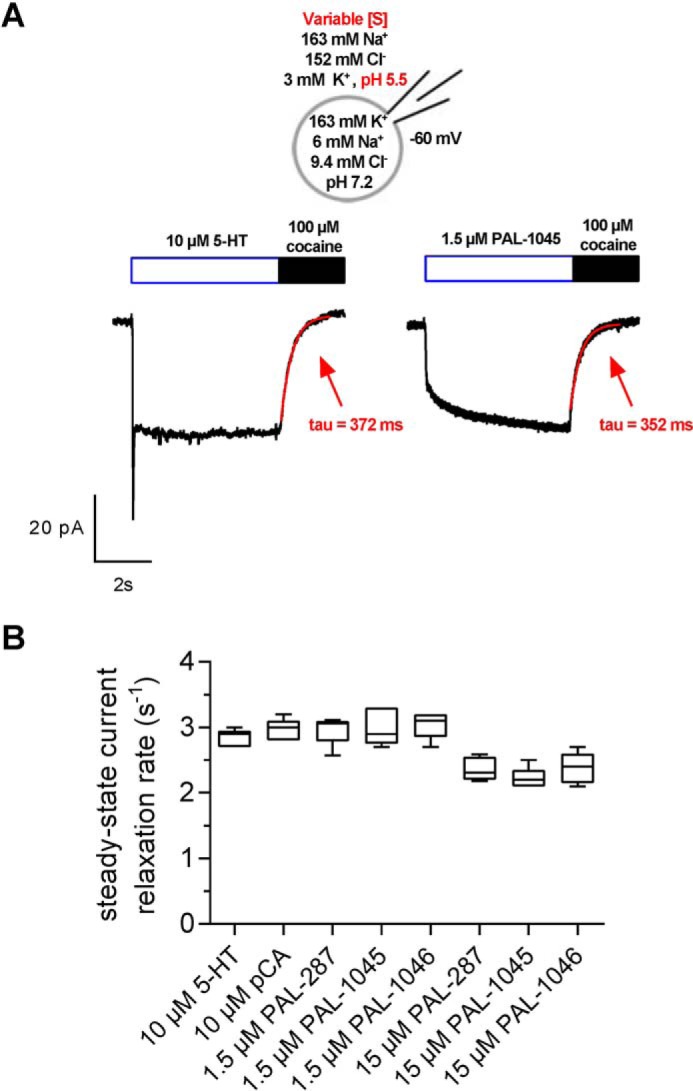
**Comparison of the steady-state relaxation rate of SERT operating in the forward transport mode in the presence of 5-HT, pCA, PAL287, PAL1045, and PAL1046.** *A*, schematic rendering of the experimental conditions (*right-hand scheme*) and representative traces of substrate-induced steady-state currents induced by 10 μm 5-HT and 1.5 μm PAL1045 in an external solution of pH of 5.5 (to limit internal accumulation and substrate rebinding) and at holding potentials of −60 mV. Substrate application was terminated by the switch to a solution containing 100 μm cocaine. This ensured occupancy of all external open binding sites of SERT by cocaine. At this (high) cocaine concentration, the ensuing relaxation of the steady-state current is not limited by the rate of cocaine binding; instead, the time course represents the sum of all partial reactions from the dissociation intracellular substrate and co-substrate dissociation to K^+^ dissociation from the outward open state of the transporter. Fitting a monoexponential function to the steady-state relaxation gives the steady-state relaxation rate *k*_r_ and its inverse (τ = 1/*k*_r_). *B*, the values of *k*_r_ were determined as shown in *panel A* for each individual compound at the indicated concentration(s). *Box plots* illustrate median values and interquartile ranges; *whiskers* display minimum and maximum values; the values for *k*_r_ were (mean ± S.D.; *n* = 5 cells per condition): 10 μm 5-HT = 2.84 ± 0.13 s^−1^, 10 μm pCA = 2.96 ± 0.17 s^−1^, 1.5 μm PAL287 = 2.96 ± 0.22 s^−1^, 1.5 μm PAL1045 3.00 ± 0.28 s^−1^, 1.5 μm PAL1046 = 3.04 ± 0.20 s^−1^, 15 μm PAL287 = 2.36 ± 0.18 s^−1^, 15 μm PAL1045 = 2.22 ± 0.16 s^−1^, and 15 μm PAL-1046 = 2.38 ± 0.23 s^−1^.

Although the absence of Na^+^ lowered the affinity of PAL287 and of PAL1045, the drop in affinity was too small to account for the discrepancy between their potency in inhibiting uptake and in blocking binding (Table 1). In addition, tight binding to the inward-facing conformation is predicted to result in a slowdown of the transport cycle, which ought to affect *V*_max_ to a larger extent than the 25–35% reduction in steady-state current seen in Fig. 2*D*. Accordingly, we investigated the internal dissociation rate of the three compounds. This is indirectly accessible to measurements; substrate-induced steady-state currents represent the proportion of SERT moving through the transport cycle in the forward mode (22). Upon substrate removal, the steady-state current decays to baseline (Fig. 5*A*). The time constant derived by fitting a monoexponential curve to this current decay reflects all partial reactions associated with dissociation of substrate and co-substrate from the inward-facing state, the subsequent binding of potassium, and the return of the potassium-bound transporter to the outward-facing conformation. This latter step is the rate-limiting step of the transport cycle and proceeds with a rate of 2 to 3 s^−1^ (18, 21). If the naphthylpropane-2-amines, in particular PAL1045, dissociated from the inward-facing conformation with the rate constants calculated for external dissociation in the presence of 10 mm external Na^+^ or in the absence of internal Na^+^ (see Fig. 5), the decay of the steady-state current would be substantially delayed. Accordingly, we applied the substrates under conditions that supported the forward transport mode (*i.e.* high internal K^+^, low internal Na^+^), and we reduced the external pH to 5.5 to limit diffusion of PAL287, PAL1045, and PAL1046 (*cf.* Fig. 2). In addition, we obviated their possible rebinding by exchanging the substrate solution for a solution containing a saturating concentration of cocaine (100 μm). PAL287, PAL1045, and PAL1046 were applied at 1.5 μm (a concentration at which >90% *V*_max_ of PAL steady-state currents are achieved; *cf.* Fig. 2*E*), whereas 5-HT and pCA were applied at 10 μm. As shown in Fig. 6*A*, the decay rates of the steady-state currents were similar for steady-state currents supported by serotonin or by PAL1045. This was also true for the other compounds with relaxation rates in the range of ∼2.5 to 3 s^−1^ (Fig. 6*B*). These observations imply that the dissociation of all compounds including PAL287, PAL1045, and PAL1046 from the inward-facing state of SERT is not slower than the rate-limiting return step of the SERT transport cycle, *i.e.* the K^+^-bound, substrate-free transporter flipping back from inward-facing to the outward-facing conformation. We also tested PAL287, PAL1045, and PAL1046 at a higher concentration (15 μm); this resulted in a modest decrease in the relaxation rate, which we attributed to diffusion of the residual non-protonated fraction of the compounds through the cell membrane and subsequent binding to the inward-facing transporter. Thus, although PAL287, PAL1045, and PAL1046 differ markedly in their affinities for the outward open state in the presence of high Na^+^ concentrations (Figs. 1*C* and 4*B* and Table 1), they have similar dissociation rates from the inward-facing conformation. This dissociation rate must be faster than determined at low Na^+^ or in the absence of Na^+^.

### Pharmacochaperoning action of PAL1045

**Figure 7.**
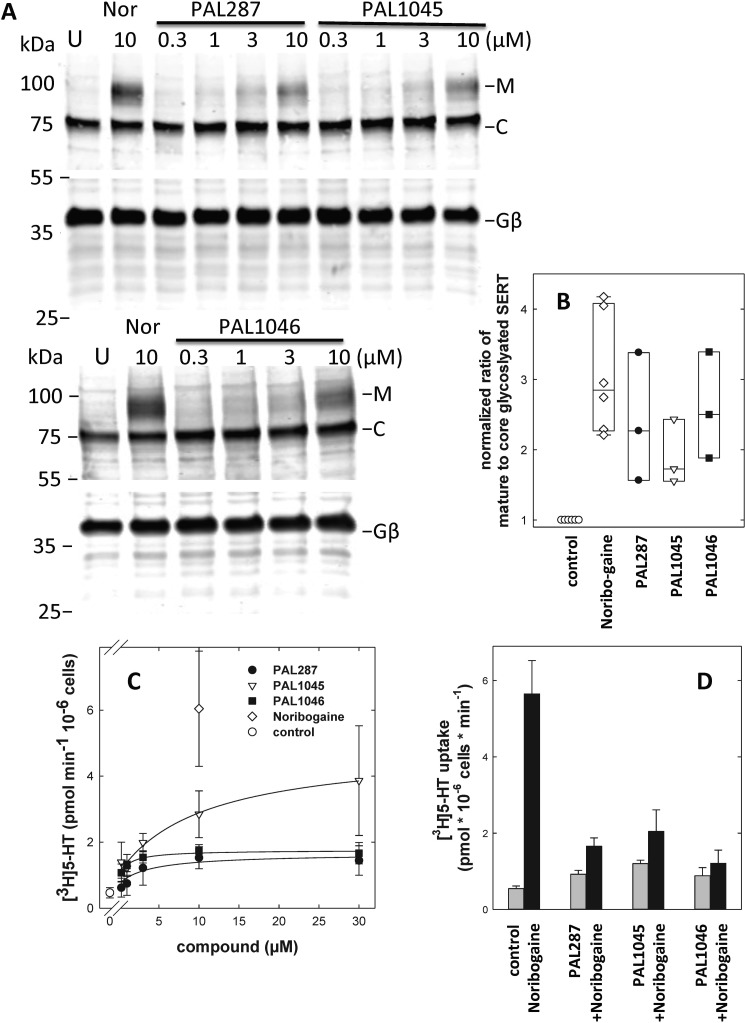
**Enhanced mature glycosylation of and substrate transport by SERT-^601^PG^602^-AA after preincubation of HEK293 cells in the absence of noribogaine, PAL287, PAL1045, and PAL1046 the cells compounds.** *A*, confluent cultures of HEK293 cells stably expressing SERT-^601^PG^602^-AA (10 cm dish/condition) were treated with either noribogaine (*Nor*; 10 μm) or PAL287, PAL1045, and PAL1046 in the indicated concentrations for 24 h. Untreated cells were taken as negative controls (*first lane* labeled *U* in each blot). Membrane proteins extracted from these cells were denatured and resolved with SDS-PAGE. After transfer of the proteins onto nitrocellulose membranes, the blots were incubated overnight at 4 °C with anti-GFP (*top*) or anti-G_β_ (*bottom*, loading control) antibody. The immunoreactive bands were detected with fluorescently labeled secondary antibody. The blot is representative of three independent experiments. *B*, the ratio of mature (*M*) to core glycosylated band (*C*) was quantified densitometrically and compared with the ratio observed in each blot for untreated control cells, which was set 1. Shown are the individual values for each compound at 10 μm. The *box plot* sows the median and the interquatrile range; *whiskers* indicate the 5–95% confidence interval. The difference between untreated cells and all other treatments was statistically significant (*p* < 0.05, Friedman test). *C*, HEK293 cells stably expressing SERT-^601^PG^602^-AA mutants were seeded onto 48-well plates and treated either with 10 μm noribogaine as a positive control or with indicated concentrations of PAL287, PAL1045, and PAL1046 for 24 h. Untreated cells were taken as controls (○). Specific [^3^H]5-HT uptake (0.2 μm) was assayed as outlined under “Experimental Procedures.” Substrate accumulation in presence of 10 μm paroxetine was used to determine nonspecific uptake. The data were obtained from three independent experiments carried out in triplicate. The *error bars* indicate S.D. The lines were drawn by fitting the data to the equation of a rectangular hyperbola. The EC_50_ values were 6.0 ± 1.8, 10.9 ± 4.4, and 1.4 ± 0.4 μm for PAL287, PAL1045, and PAL1046, respectively. *D*, HEK293 cells stably expressing SERT-^601^PG^602^-AA mutants were incubated in the absence (*control*) and presence of 10 μm noribogaine, 2.5 μm PAL287, 2.5 μm PAL1045, and 2.5 μm PAL1046 or the combination of noribogaine and PAL287, PAL1045, or PAL1046 for 24 h. Thereafter, specific [^3^H]5-HT uptake (0.2 μm) was determined. Data are from three independent experiments carried out in triplicate. The *error bars* indicate S.D. The differences between noribogaine and the combination of noribogaine with any of the PAL compounds was statistically significant as was the difference between control and any of the PAL compounds (*p* < 0.01 analysis of variance followed by post hoc comparison by a Holm-Sidak test).

**Figure 8.**
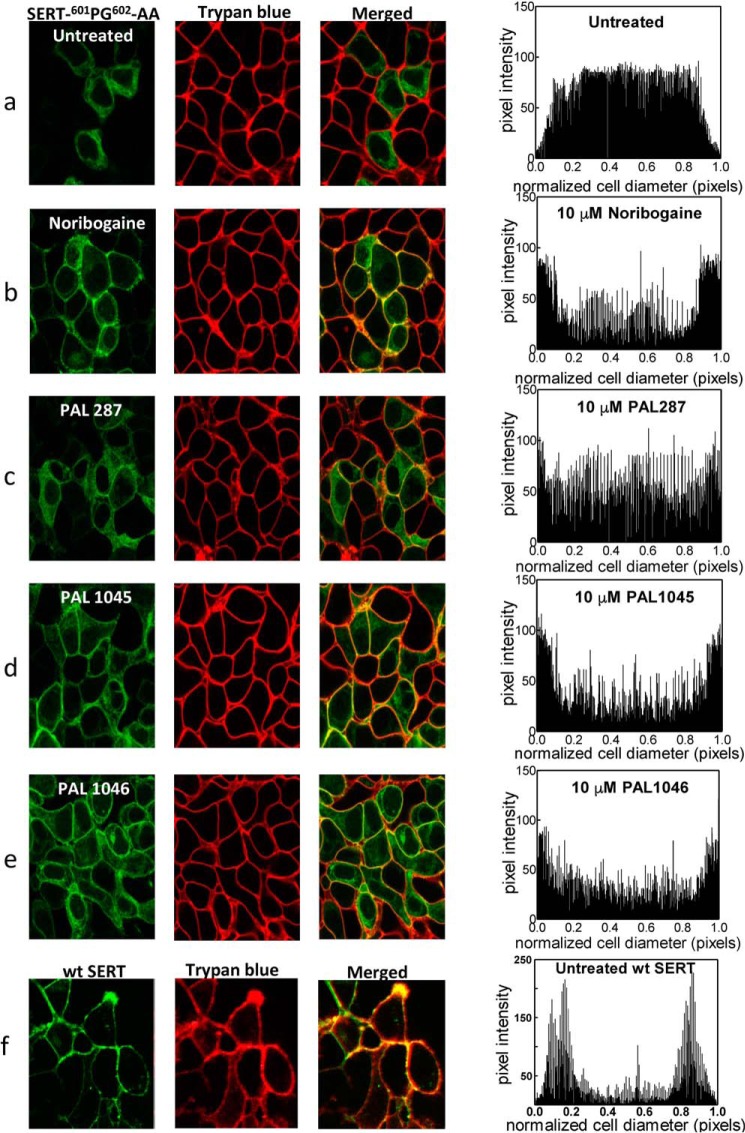
**Cellular distribution of YFP-tagged SERT-^601^PG^602^-AA in HEK293 cells, which had been incubated in the absence and presence of noribogaine, PAL287, PAL1045, and PAL1046 and of YFP-tagged wild-type SERT stably expressed in HEK 293 cells.** HEK293 cells stably expressing YFP-tagged SERT-^601^PG^602^-AA mutant were seeded onto poly-d- lysine-coated ibidi® glass-bottom chambers and incubated in medium (*a*) or treated with 10 μm noribogaine (*b*), 10 μm PAL287 (*c*), 10 μm PAL1045 (*d*), and 10 μm PAL1046 (*e*) 24 h before confocal imaging. Confocal images were captured on a Zeiss LSM710 microscope to visualize YFP-tagged SERT-^601^PG^602^-AA SERT (shown in the *green channel*) and trypan blue (0.05% in PBS, shown in the *red channel*) to label the plasma membrane. In the *bottom row* (*f*), the distribution of wild-type SERT in HEK293 cells was visualized for comparison. Channels were merged to show co-localization between the signals. In the *right-hand column*, the distribution of the SERT-^601^PG^602^-AA mutant and wild-type SERT was quantified: signal distribution (pixel intensity) was measured over cellular cross-sections using ImageJ in >10 cells per condition (in three independent experiments) and plotted in a cumulative manner.

Taken together the experiments summarized in Figs. 2–6 explain why the affinity of PAL287, PAL1045, and PAL1046 differ under various conditions. Importantly, these observations also highlight the fact that the compounds, in particular PAL1045, bind to several conformations of SERT with high affinity. We, therefore, surmised that these compounds also ought to be suitable to promote folding of the transporter because they may bind to and stabilize folding intermediates and thus smooth the energy landscape of the folding trajectory. We verified this conjecture by selecting the folding-deficient mutant SERT-^601^PG^602^-AA; the folding defect is also evident from Fig. 7*A*, because essentially all of SERT-^601^PG^602^-AA was detected as an endoplasmic reticulum-retained, core-glycosylated band (labeled *C*) in lysates from cells, which had been maintained in the absence of any compound (*lanes* labeled *U* in Fig. 7*A*). If cells were incubated in the presence of noribogaine, a mature-glycosylated band appeared (labeled *M*, *cf. lanes 1* and *2* in Fig. 7*A*). We verified that these bands differed in their sensitivity to endoglycosidase H (not shown, see Ref. 14). Incubation of cells with increasing concentrations of PAL287, PAL1045, and to a lesser extent PAL1046 resulted in the appearance of the mature glycosylated band of SERT-^601^PG^602^-AA (Fig. 7, *A* and *B*). We verified that this mature glycosylated transporter was (i) functional by measuring substrate uptake (Fig. 7*C*) and (ii) delivered to the cell surface (Fig. 8). The concentration-response curve illustrated in Fig. 7*C* documented that PAL1045 was the most efficacious pharmacochaperone of the three compounds. Because the pharmacochaperoning effect was determined in a stably transfected cell line, it is not possible to gauge from the experiments depicted in Fig. 7*C* how much of transport activity was recovered relative to that of wild-type SERT. However, experiments carried out in transiently transfected cells indicate that cells expressing SERT-^601^PG^602^-AA recovered ∼30 and 20% of the transport activity of wild-type SERT (not shown, see also Ref. 15). We also explored the effect of the combined addition of noribogaine and PAL287, PAL1045, or PAL1046. As can be seen from Fig. 7*D*, PAL287, PAL1045, and PAL1046 antagonized the rescue elicited by noribogaine. This observation proves that noribogaine and the PAL-compounds compete for the same binding site. In addition, this observation indicates that a pharmacochaperone must not only bind but also display an additional “intrinsic” activity in a manner analogous to the action of partial and full agonists on a receptor.

In the absence of any pretreatment, SERT-^601^PG^602^-AA was mainly visualized within the cell by confocal microscopy, and there was no appreciable colocalization with the trypan blue fluorescence, which was used to delineate the cell surface (*top row* in Fig. 8*a*). In contrast, if cells had been incubated in the presence of noribogaine (*second row*), PAL287 (*third row*), PAL1045 (*fourth row*), and PAL1046 (*bottom row*), there was substantial colocalization of SERT-^601^PG^602^-AA with the trypan blue stain in the plasma membrane. The pharmacochaperone-induced appearance of SERT-^601^PG^602^-AA was also evident from the cumulative distribution of the fluorescence intensity shown in Fig. 8*b*.

## Discussion

The transport cycle of SERT is understood in considerable detail (5, 18, 21); when moving through the transport cycle in the forward transport mode, SERT undergoes a series of conformational transitions, the kinetics of which are accessible via electrophysiological recordings. The outward-facing conformation of SERT can be probed independently by using radiolabeled inhibitors, and the global turnover rate of SERT can be estimated by measuring the rate of substrate uptake. We employed a combination of these approaches to understand why the naphthylpropane-2-amine PAL1045 was a “partial” substrate (9) and to confirm that these unique properties made PAL1045 suitable for pharmacochaperoning. PAL1045 differs substantially from the endogenous substrate serotonin or the prototypical exogenous substrate *p*-chloroamphetamine because, first, it binds with very high affinity to the transporter. In fact, the estimate for the dissociation constant of 5 nm is comparable to that of therapeutically relevant SERT inhibitors (24). Our observations showed that this high affinity was due to a very slow dissociation rate and that, consistent with typical SERT inhibitors, the dissociation from the transporter was dependent on the Na^+^ concentration (25). Second, however, in contrast to typical SERT inhibitors, which trap SERT in the outward-facing conformation, PAL1045 is still a substrate. This conclusion is based on our analysis of the steady-state currents, which were effectively elicited by PAL1045 and by its congeners provided that their intracellular accumulation was prevented by lowering the extracellular pH to 5.5. Third, in contrast, when PAL1045 and its congeners were applied at pH 7.4, they accumulated within the cell to an extent that blocked the forward transport mode. This provided indirect evidence for binding of PAL1045 to the inward-facing conformation of SERT. Binding to the inward-facing conformation was also evident from the time course of peak current recovery recorded in the absence of intracellular Na^+^. Taken together, these observations confirmed that PAL1045 bound to several conformational states with appreciable affinity, *i.e.* the dwell time of the compound within the binding pocket(s) of the transporter ought to be long enough to result in pharmacochaperoning activity. This prediction was confirmed in three independent approaches with the mutant SERT-^601^PG^602^-AA. Of the several available folding mutants of SERT, this is the most affected (14–16). In fact, the pharmacochaperoning activity of PAL1045 approached that of noribogaine. Its congeners PAL287 and PAL1045 also had appreciable pharmacochaperoning activity, but they were less efficacious than PAL1045.

It is not clear how the folding trajectory of SERT proceeds. The available, albeit circumstantial evidence suggests that it moves through the inward-facing conformation because ibogaine and/or its derivative noribogaine rescue the folding defect of all SERT mutants so far examined (14–16); ibogaine traps SERT in the inward-facing conformation (12, 13). In addition, mutations, which stabilize SERT in the inward-facing conformation such as SERT-E136A (25) and SERT-T81A (26), act as second-site suppressors and restore cell-surface expression of folding-deficient SERT mutants (15). The folding trajectory can be envisaged as a conformational search through a rugged multidimensional space littered with energy barriers (27). In fact, individual SERT mutants are stalled at different stages of the folding trajectory such that they differ in their susceptibility to second-site suppressors (15). The binding modes of PAL1045 and of noribogaine are distinct; it is, therefore, conceivable that in the folding trajectory PAL1045 lowers an energy barrier other than that affected by noribogaine. The interest in the search for pharmacochaperones has been fueled by the discovery of folding-defective mutants of SLC6 transporters, which give rise to diseases in people (11, 28). There is, in particular, a long list of point mutations of the gene encoding the human dopamine transporter, which give rise to a syndrome of infantile/juvenile dystonia and Parkinsonism; the vast majority of the resulting mutant versions of DAT are dysfunctional because they are defective in folding (29–31). Some of these mutants are amenable to rescue by noribogaine and bupropion (32), but many are not.^4^ Accordingly, it appears reasonable to assume that many different compounds will be required if all folding defects are to be remedied. The fact that these can be identified by analyzing their impact on the transport cycle is reassuring; monoamine transporters have a very rich pharmacology (5). Hence, we are confident that it will be possible to identify many additional pharmacochaperones by focusing on compounds with atypical properties. In addition, a large collection of pharmacochaperones in combination with a long list of folding-deficient mutants is likely to provide a platform for addressing the folding trajectory of SLC6 transporters.

Our experiments produced several surprising findings. (i) The association rate constant of *p*-chloroamphetamine was 7-fold smaller than that of serotonin and of the three naphthylpropane-2-amines. These observations are somewhat counterintuitive because both serotonin and the naphthylpropane-2-amines are larger in size than *p*-chloroamphetamine. However, it was noted previously that inhibitors can differ substantially in their bimolecular rate constant despite related sizes. In fact, it was the association rate rather than the dissociation rate that explained the selectivity of inhibitors for SERT over NET and vice versa (20). One explanation is that the outer vestibule of monoamine transporters acts in a manner equivalent to a selectivity filter. In this hypothetical model various docking poses of *p*-chloroamphetamine are subject to triage in the access pathway to the binding site proper. In contrast, the access of PAL287, PAL1045, PAL1046, and serotonin are impeded to a substantially lesser extent. (ii) The Na^+^-dependent dissociation rate of PAL1045 differed substantially, *i.e.* by 5-fold, from that of PAL287 and PAL106. It is known that SERT prefers tertiary amines (*e.g.* imipramine, amitryptyline) over the corresponding secondary amines (desipramine, nortriptyline) (24). Although this can account for the differences in affinity between PAL1045 and PAL287, it cannot explain why PAL1046 has substantially lower affinity than PAL1045. The additional methyl group in PAL1045 may preclude the solvation of the binding pocket and hence reduce the movement of Na^+^. (iii) The tight binding of PAL1045 was not rate-limiting for the return step; upon superfusion with cocaine, the steady-state current elicited by PAL1045 decayed as rapidly as that induced by serotonin. The rate (of ∼2.8 s^−1^) is substantially faster than that of the dissociation rate of PAL1045 from the inward-facing conformation in the nominal absence of intracellular Na^+^ and K^+^. Thus, there must be a reaction that accelerates the dissociation of substrates including PAL1045 from the inward-facing state. In fact, binding of K^+^ to the inward-facing state is crucial to drive the forward transport mode of SERT (21). We, therefore, suspect that binding of K^+^ does not only cancel out the electrogenic release of Na^+^ from the inward-facing conformation but also accelerates substrate release. This hypothesis is currently being addressed. Regardless of the underlying cause, rapid dissociation of PAL1045 from the inward-facing conformation also accounts for the observations that the concentrations required for pharmacochaperoning were in the micromolar range. In contrast, binding to the Na^+^-induced outward-facing state occurred in the nanomolar concentration range, *i.e.* the concentrations differed by 3 orders of magnitude. It is difficult to estimate the steady-state ionic composition in the lumen of the endoplasmic reticulum with reasonable precision because there are large fluxes of Ca^2+^ that are buffered by many lumenal proteins and exchanged for by counter-movements of K^+^ and H^+^ (33). Nevertheless, it is clear that the Na^+^ concentration in the lumen of the endoplasmic reticulum must be trivial. Hence, it is unlikely that Na^+^-stabilized outward-facing conformations are relevant in the folding trajectory of SERT or of any other SLC6 transporter.

Monoamine transporters are closely related. PAL1045 has appreciable affinity for DAT (9). We are, therefore, confident that PAL1045 and related compounds will expand the list of useful pharmacochaperones. There are two features that make PAL1045 attractive; if SERT is allowed to cycle in the forward transport mode, it is only a low affinity inhibitor. In addition, it is only a partial releaser (9). Hence, it is less likely to cause amphetamine-like addiction. At the very least it can be argued that PAL1045 provides a useful lead to search for compounds with enhanced pharmacochaperoning activity in a rational manner.

## Experimental procedures

### Materials

Cell culture media and antibiotics were obtained from Sigma and InvivoGen, respectively. [^3^H]5-HT, serotonin, 23.9 Ci/mmol), and [^3^H]imipramine (51.6 Ci/mmol) were purchased from PerkinElmer Life Sciences. Scintillation mixture (Rotiszint® eco plus) was from Carl Roth GmbH (Karlsruhe, Germany). Anti-GFP antibody (rabbit, ab290) was from Abcam (Cambridge, UK). An antiserum directed against the N terminus of G protein β_1_ and β_2_ subunits (34) was used to verify comparable loading of lanes. The secondary antibody (Donkey anti-rabbit, IRDye 680RD) was obtained from LI-COR Biotechnology GmbH (Bad Homburg, Germany). All other chemicals were of analytical grade. Noribogaine was purchased from Cfm Oskar Tropitzsch GmbH (Marktredwitz, Germany).

### [^3^H]5-HT uptake assays

For uptake assays HEK293 cells stably expressing wild-type human YFP-tagged SERT were seeded on poly-d-lysine-coated 48-well plates at a density of ∼10^5^ cells/well. After 24 h the medium was removed, the cells were washed in Krebs-Hepes buffer (10 mm HEPES·NaOH, pH 7.4, 120 mm NaCl, 3 mm KCl, 2 mm CaCl_2_, 2 mm MgCl_2_, and 2 mm glucose) and subsequently incubated for 1 min in the presence of 0.2 μm [^3^H]5-HT and logarithmically spaced concentrations (0.3–100 μm) of *p*-chloroamphetamine, PAL287, PAL1045, or PAL1046. Nonspecific uptake was defined in the presence of 10 μm paroxetine. After 1 min the reaction was terminated by aspiration of the reaction medium followed by washes with ice-cold buffer. The cells were then lysed with 1% SDS to release the retained radioactivity, which was quantified by liquid scintillation counting. Before the determination of uptake velocity, HEK293 cells stably expressing YFP-tagged SERT-^601^PG^602^-AA (14) were seeded as mentioned above and incubated in the absence or presence of 10 μm noribogaine, 10 μm *p*-chloroamphetamine, or of increasing concentrations (0.3, 1, 3, 10 μm) of PAL287, PAL1045, or PAL1046. After 24 h the cells were washed with Krebs-HEPES buffer and subsequently incubated with [^3^H]5-HT (0.2–30 μm) for 1 min as outlined above.

### [^3^H]Imipramine binding

Membranes were prepared from HEK293 cells stably expressing human GFP-tagged SERT as outlined previously (26). The binding reaction was carried out in a final volume of 0.2 ml of buffer (20 mm Tris-HCl, pH 7.4, 1 mm EDTA, 2 mm MgCl_2_, 120 mm NaCl, 3 mm KCl), membranes (2.5 μg/assay), [^3^H]imipramine (3 nm), and the logarithmically spaced concentrations of *p*-chloroamphetamine (0.1–100 μm), PAL287 (0.03–10 μm), PAL1045 (0.3–300 nm), or of PAL1046 (3 nm to 3 μm) at 20 °C for 1 h. The binding reactions were terminated by harvesting the membranes on glass fiber filters precoated with polyethyleneimine and rapid washing with ice-cold wash buffer (10 mm Tris·HCl, pH 7.4, 120 mm NaCl, 2 mm MgCl_2_). The radioactivity trapped on the filters was quantified by liquid scintillation counting. Nonspecific binding was defined in the presence of 10 μm paroxetine.

### Whole-cell patch clamp recordings

HEK293 cells stably expressing wild-type GFP-tagged human SERT were seeded at low density on poly-d-lysine-coated dishes. Twenty-four hours after seeding these cells were subjected to patch clamp recordings in the whole cell configuration; we recorded substrate-induced transient peak and steady-state currents carried through SERT as outlined previously (18, 21). In most instances cells were continuously maintained in an external solution containing 140 mm NaCl, 3 mm KCl, 2.5 mm CaCl_2_, 2 mm MgCl_2_, 20 mm glucose, and 10 mm HEPES (external solution 1, pH adjusted to 7.4 with NaOH). In some instances the following changes were made: (i) the external pH was lowered to 5.5 by substituting 10 mm HEPES 10 mm MES (methanesulfonate) as buffer (pH adjusted to 5.5 with NaOH, external solution 3); (ii) a Na^+^-free external solution was made by replacing 140 mm NaCl in external solution 1 with 140 mm NMDG chloride (pH adjusted to 7.4 with NMDG, external solution 3); (iii) external solution with 10 mm sodium concentration was obtained by mixing appropriate amounts of external solutions 1 and 3.

The internal solution in the patch pipette contained 133 mm potassium gluconate (CH_2_OH(CHOH)_4_COOK), 5.9 mm NaCl, 1 mm CaCl_2_, 0.7 mm MgCl_2_, 10 mm HEPES, 10 mm EGTA (internal solution 1, pH adjusted to 7.2 with KOH). For a Na^+^-free, K^+^-free, and high Cl^−^ internal solution, NaCl and CH_2_OH(CHOH)_4_COOK in internal solution 1 were replaced by NMDG chloride (internal solution 2, pH adjusted to 7.2 by NMDG). For a Na^+^-free, K^+^-free, and Cl^−^-free internal solution, NaCl, CH_2_OH(CHOH)_4_COOK, CaCl_2_, and MgCl_2_ of internal solution 1 were replaced by NMDG, methanesulfonate, calcium methanesulfonate, and magnesium acetate, respectively (internal solution 3, pH adjusted to 7.2 by NMDG). A sodium- and potassium-free internal solution containing 0.5 mm chloride was made by mixing internal solution 2 with internal solution 3 in a ratio of 1:285. In experiments where the internal solution contained high sodium, 133 mm CH_2_OH(CHOH)_4_COOK in internal solution 1 was replaced with 133 mm NaCl (pH adjusted to 7.2 by NaOH, internal solution 4).

Drugs were applied using a 4 tube or 8 tube ALA perfusion manifold (NPI Electronic GmbH, Germany) and a DAD-12 superfusion system (Adams & List, Westbury, NY) allowing for complete solution exchange around the cells within 100 ms. Drugs were applied for 200 ms and 5 s to obtain peak and steady-state currents, respectively. Current amplitudes and associated kinetics were quantified using Clampfit 10.2 software. Passive holding currents were subtracted, and the traces were filtered using a 100-Hz digital Gaussian low-pass filter.

### Immunoblotting and confocal microscopy after pharmacochaperoning of SERT-^601^PG^602^-AA

HEK293 cells stably expressing mutant YFP-tagged SERT-^601^PG^602^-AA (1 × 10^6^ cells/dish) were incubated in Dulbecco's modified Eagle's medium with high glucose (4.5 g/liter) and l-glutamine (584 mg/liter) containing 10% fetal calf serum and gentamicin (50 μg/ml) in the absence and presence of noribogaine (10 μm), PAL287 (0.3–10 μm), PAL1045 (0.3–10 μm) or PAL1046 (0.3–10 μm) for 24 h. Subsequently, the cells were washed ice-cold phosphate-buffered saline, detached mechanically, and harvested by centrifugation (5 min, 1000 × *g*). The cell pellet was resuspended in lysis buffer containing Tris·HCl, pH 8.0, 150 mm NaCl, 1% dodecyl maltoside, 1 mm EDTA, and protease inhibitors (Complete^TM^, Roche Applied Science). The detergent-insoluble material was removed by centrifugation (16,000 × *g* for 15 min at 4 °C). Aliquots of the supernatant (20 μg of protein) were mixed with sample buffer containing 1% SDS and 20 mm DTT, denatured at 45 °C for 30 min, and electrophoretically resolved in denaturing polyacrylamide gels. After transfer of the proteins onto nitrocellulose membranes, the blots were probed with an antibody against GFP (rabbit, ab290) at a 1:3000 dilution. The immunoreactive bands were visualized with a fluorescently labeled anti-rabbit secondary antibody (LI-COR; at 1:5000 dilution) by fluorescence imaging (Odyssey Clx, LI-COR Biosciences, Lincoln, NE) and quantified using ImageJ.

For confocal imaging, HEK293 cells stably expressing YFP-tagged SERT-^601^PG^602^-AA mutant were seeded onto poly-d-lysine-coated ibidi® glass-bottom chambers. Cells were incubated for 24 h in the absence and presence of noribogaine, PAL287, PAL1045, or PAL1046 (each at 10 μm) as outlined above. Thereafter, the medium was replaced by a trypan blue solution (0.05% trypan blue in PBS) to stain the cell surface. Confocal images were captured on a Zeiss LSM780 microscope equipped with a 63× oil immersion objective. Quantification of fluorescence intensity was done using ImageJ. Signal distribution (pixel intensity) was measured over a cellular cross-section of at least 10 cells per condition using the built-in plot profile analysis tool.

## Author contributions

M. F., P. S. H., W. S., and S. B. designed the experiments and wrote the paper. S. B. performed the experiments in Figs. 1–7, A. E.-K., M. F., and S. S. carried out supportive experiments. A. K. performed the experiments in Fig. 8. B. E. B. synthesized the PAL compounds. A. E.-K., M. H. B., and S. S. provided reagents, advice, and cell lines. All authors reviewed the results and approved the final version of the manuscript.

## Footnotes

4 S. Sucic, A. Kasture, A. El-Kasaby, and M. Freissmuth, unpublished observations.

## Footnotes

3 The abbreviations used are:

SERT: serotonin transporter
DAT: dopamine transporter
NET: norepinephrine transporter
SLC6: solute carrier family 6
5-HT: 5-hydroxytryptamine (serotonin)
GFP: green fluorescent protein
NMDG: *N*-methyl-d-glucamine
PAL: phenethylamine library
PAL287: (±)-1-(2-naphthyl) propan-2-amine
PAL1046: (*S*)-*N*-methyl-1-(2-naphthyl)propan-2-amine
PAL1045: (*S*)-*N*-ethyl-1-(2-naphthyl)propan-2-amine
pCA: *p*-chloroamphetamine
YFP: yellow fluorescent protein
CI: confidence level.
